# A Novel Pathogenic *HSPG2* Mutation in Schwartz–Jampel Syndrome

**DOI:** 10.3389/fneur.2021.632336

**Published:** 2021-03-09

**Authors:** Po-Yu Lin, Jia-Horung Hung, Chao-Kai Hsu, Yao-Tsung Chang, Yuan-Ting Sun

**Affiliations:** ^1^Department of Neurology, National Cheng Kung University Hospital, College of Medicine, National Cheng Kung University, Tainan, Taiwan; ^2^Institute of Clinical Medicine, College of Medicine, National Cheng Kung University, Tainan, Taiwan; ^3^Department of Ophthalmology, National Cheng Kung University Hospital, College of Medicine, National Cheng Kung University, Tainan, Taiwan; ^4^Department of Dermatology, National Cheng Kung University Hospital, College of Medicine, National Cheng Kung University, Tainan, Taiwan; ^5^Department of Genomic Medicine, National Cheng Kung University Hospital, College of Medicine, National Cheng Kung University, Tainan, Taiwan; ^6^Institute of Basic Medical Sciences and Department of Biochemistry and Molecular Biology, National Cheng Kung University College of Medicine, Tainan, Taiwan

**Keywords:** Schwartz–Jampel syndrome, heparan sulfate proteoglycan 2, perlecan, short exercise test, primary fibroblast

## Abstract

Schwartz–Jampel syndrome is a rare autosomal recessive disease caused by mutation in the heparan sulfate proteoglycan 2 (*HSPG2*) gene. Its cardinal symptoms are skeletal dysplasia and neuromuscular hyperactivity. Herein, we identified a new pathogenic mutation site (NM_005529.6:c.1125C>G; p.Cys375Trp) of *HSPG2* leading to Schwartz–Jampel syndrome by whole-exome sequencing. This mutation carried by the asymptomatic parents was previously registered in a single-nucleotide polymorphism database of the National Institutes of Health as a coding sequence variant rs543805444. The pathogenic nature of this missense mutation was demonstrated by *in silico* pathogenicity assessment, clinical presentations, and cellular function of primary fibroblast derived from patients. Various *in silico* software applications predicted the mutation to be pathogenic [Sorting Intolerant From Tolerant (SIFT), 0; Polyphen-2, 1; CADD (Combined Annotation Dependent Depletion), 23.7; MutationTaster, 1; DANN (deleterious annotation of genetic variants using neural networks); 0.9]. Needle electromyography revealed extensive complex repetitive discharges and multiple polyphasic motor unit action potentials in axial and limb muscles at rest. Short exercise test for myotonia showed Fournier pattern I. At cellular levels, mutant primary fibroblasts had reduced levels of secreted perlecan and impaired migration ability but normal capability of proliferation. Patients with this mutation showed more neuromuscular instability and relatively mild skeletal abnormality comparing with previously reported cases.

## Introduction

Schwartz–Jampel syndrome is a rare autosomal recessive disease with a prevalence of <1/10^6^. Its cardinal symptoms are skeletal dysplasia and neuromuscular hyperactivity. Schwartz–Jampel syndrome is caused by a mutation in the *heparan sulfate proteoglycan 2* (*HSPG2*) gene, which encodes the core protein of perlecan. Perlecan is a large multidomain proteoglycan that binds to and cross-links many extracellular matrix components. As a key component of the extracellular matrix and basement membrane, it helps to maintain muscle bulk homeostasis (1, 2), acetylcholinesterase content at the motor endplate (3), excitability at the nerve terminal (4), and muscle membrane (5). Herein, we identified a novel homozygous mutation c.1125C>G on *HSPG2* causing a p.Cys375Trp change in domain II of the perlecan core protein from two familial cases. This mutation site is first reported as a pathogenic mutation. The cellular function of primary cells derived from the patients was compared with that of normal subjects.

## Materials and Methods

### Ethics Approval

The clinical investigations were conducted according to the principles expressed in the Nuremberg Code and Declaration of Helsinki. The protocol was approved by the Institutional Review Board of National Cheng Kung University Hospital (IRB Approval No. A-BR-104-052, No. A-BR-108-055). Human subjects involved had legal capacity to give voluntary consent. Physicians had endeavored to disclose the research-relevant information to the subjects. Subjects who agreed to participate signed an informed consent form acknowledging that agreement.

### Case Description

**Figure 1:**
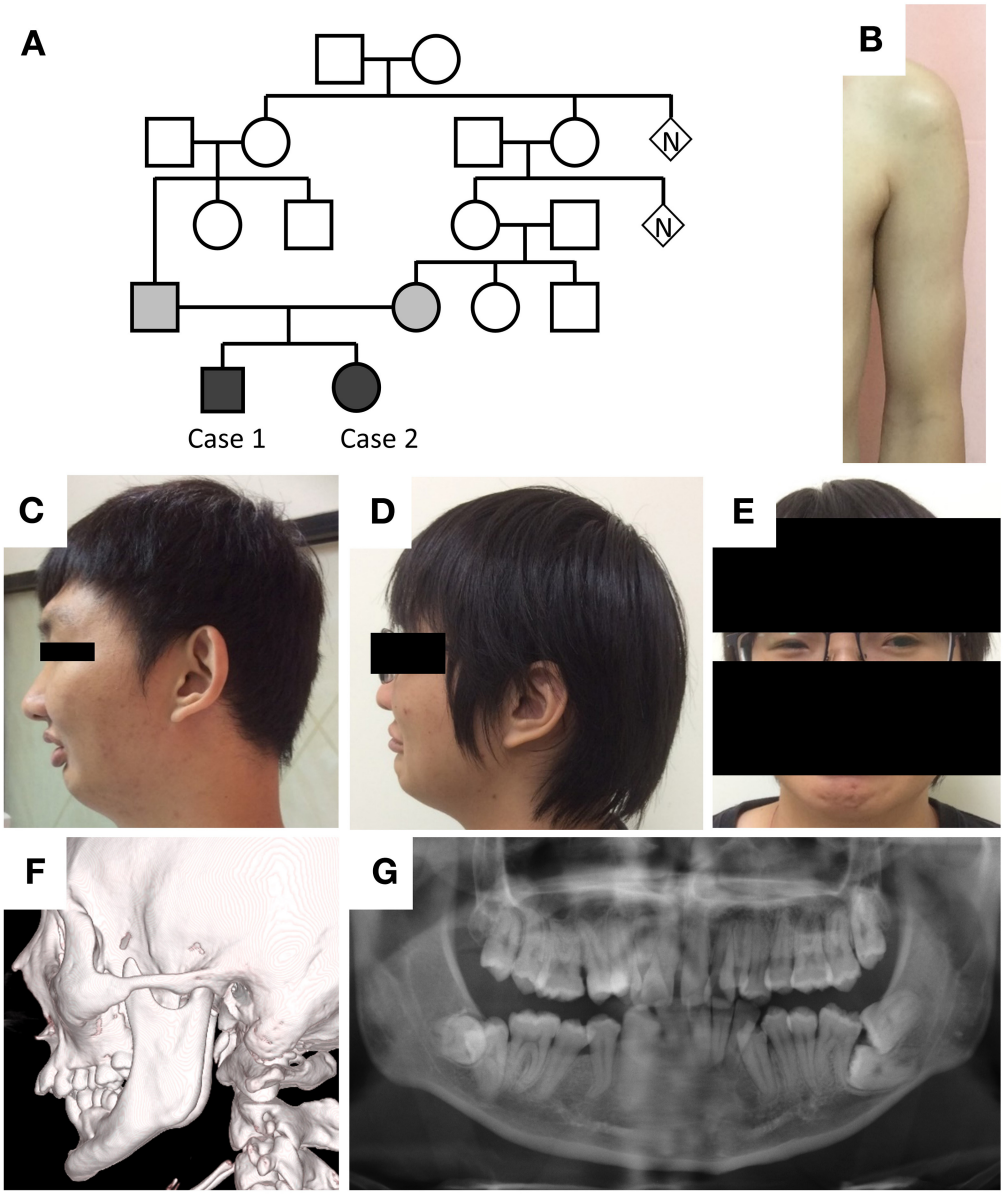
Phenotypes of the cases. **(A)** Pedigree of the two cases. **(B)** Hypertrophic pectoralis major and biceps brachii with thin subcutaneous tissue in case 1. **(C,D)** Retruded mandible, pursed lip, and supraorbital ridge in both cases. **(E)** Blepharophimosis in case 2. **(F,G)** Elongation of the bilateral coronoid processes and malocclusion in case 1.

The two cases are children of a Taiwanese family with consanguineous marriage (Figure 1A). The older man was 30 years old and presented with limited mouth opening due to hyperactivity of the jaw muscle. He frequently experienced painful muscle cramps following prolonged sitting, standing, walking, or running. The cramping would last for a few minutes and resolve spontaneously. He had poor physical endurance since childhood and was usually on the final list of grades in running contests longer than 400 m. Despite poor physical performance, he had robust muscle bulk in the proximal upper limbs (Figure 1B). Additionally, he had a retruded jaw with class II malocclusion in both primary and permanent teeth as well as poor visual acuity due to bilateral ectopia lentis.

On examination, the patient's height was 178 cm, and body weight was 80 kg. He had a retruded mandible, a prominent supraorbital ridge (Figure 1C), a normal testicular size, and hypertrophy in the bilateral biceps brachii muscles. His muscle strength and deep tendon reflexes were normal. His joints had normal range of motion. Jaw jerk and Babinski reflexes were absent. No percussion myotonia was noted in the tongue or thenar muscles. Eye examination showed irregular left pupil configuration, bilateral trace light reflex, and ectopia lentis. Pupil dilatation response to mydriatic medication was less than that observed in healthy people. His fundi were normal, and the interpalpebral distance was within normal limits.

Laboratory test showed mildly elevated serum creatine kinase levels (473 U/L). The levels of serum sodium, potassium, calcium, lactate, lactate dehydrogenase, thyroid hormone, and morning cortisol were all within normal limits. Human immunodeficiency virus screen and dry blood α-glucosidase tests were negative. Facial bone computed tomography showed elongation of the bilateral coronoid processes and excessive overjet of teeth (Figure 1F). Whole-body long-bone x-ray revealed minimal scoliosis and mild intervertebral space narrowing in L1–2 and L2–3. Echocardiography and 24-h Holter recording did not reveal cardiac abnormality.

**Figure 2:**
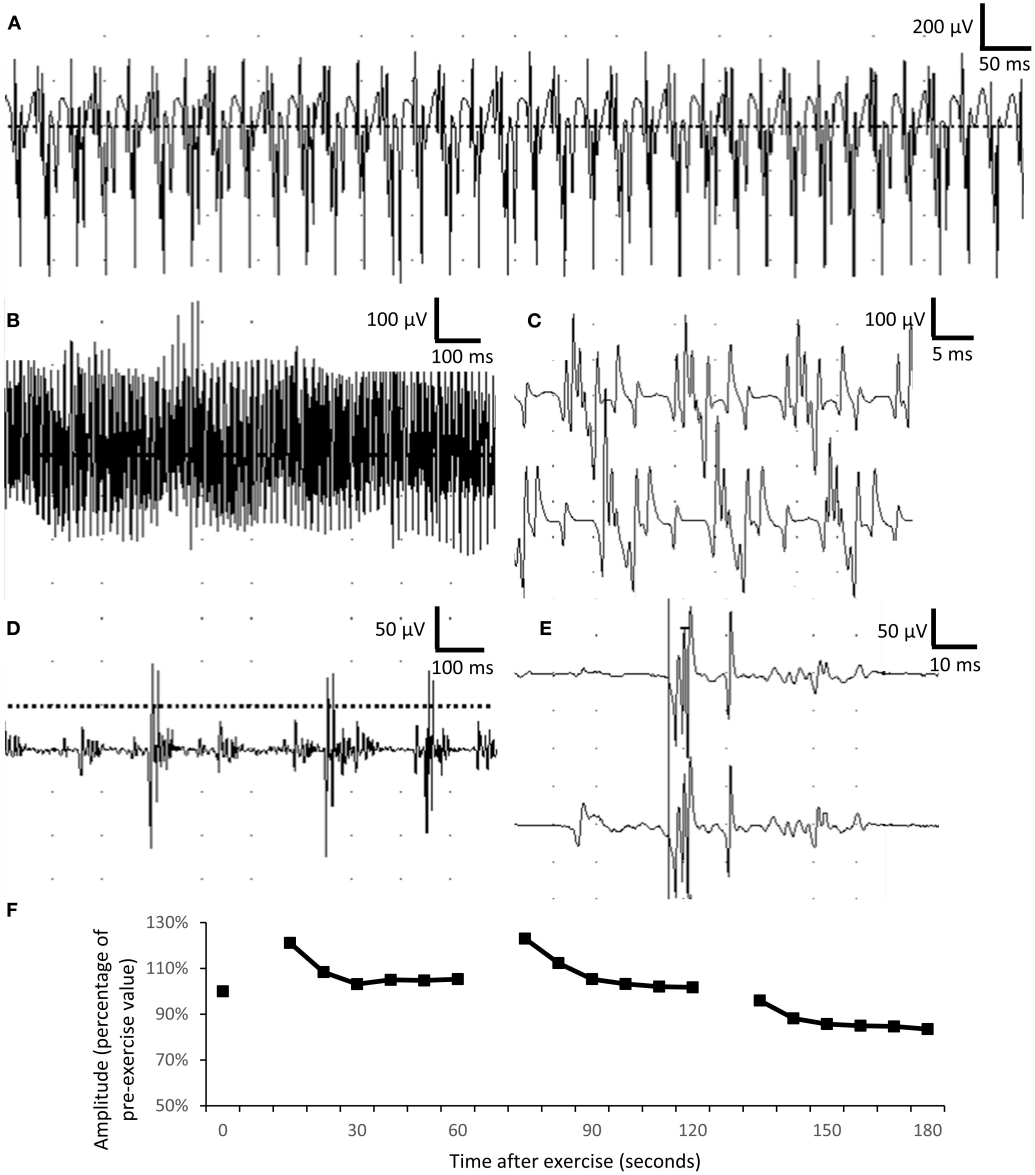
Electrophysiological studies. **(A–C)** Complex repetitive discharges were recorded in the biceps brachii. **(D,E)** Long-duration, polyphasic motor unit action potential suggesting neuropathic changes in the biceps muscle. The duration of motor unit action potential was 5–15 ms in normal muscle, comparing with more than 50-ms duration recorded in the patient. **(F)** Short exercise test for myotonia in the abductor digiti minimi showed progressively mild decremental change in motor unit action potential in three consecutive phases, compatible with Fournier pattern I.

Regarding electrophysiological studies in the peripheral nervous system, the needle electromyography (EMG) revealed extensive complex repetitive discharges (CRDs) in the axial and limb muscles in the resting phase (Figures 2A–C). Multiple polyphasic motor unit action potentials (MUAPs) with significant increase in phases and duration (Figures 2D,E) were found. For a repetitive appeared MUAP, the variation in shape and interelement latencies were scarcely detected. The presence of CRDs suggested hyperexcitability of the muscle membrane. The long duration of MUAPs suggested dispersion of conduction velocity in terminal axon branches or neuromuscular junctions. In this case, the long duration of MUAPs is more likely due to problems at terminal axon branch because little variations in shape and duration were found between repetitive fired MUAPs. Short exercise test for myotonia in the abductor digiti minimi showed progressive decrement in the amplitude of compound motor action potential in three consecutive phases, compatible with Fournier pattern I, which was known to be related to sodium-channel dysfunction (Figure 2F).

The younger woman was 27 years old and presented with intermittent muscle cramping, proximal muscle hypertrophy, retruded jaw with malocclusion, blepharophimosis (Figures 1D,E), and left intraocular lens subluxation. Her nerve conduction velocity of the four limbs was within the normal limits, and repetitive nerve stimulation test at a frequency of 3 Hz showed no decremental change. Her whole-body long-bone X-ray was also unremarkable. The two siblings had uneventful births and developmental history.

### Genetic Analysis

The next-generation sequencing was performed by the laboratory of GenePhile Bioscience (Taiwan). To generate standard exome capture libraries, SureSelect XT Human All Exon Clinical Exome version 2 (CREv2, 66Mb) probe set was used (Agilent Technology, USA). For DNA library construction, 1 μg genomic DNA was used with Agilent SureSelect XT Reagent kit. The amplification adapter-ligated sample was purified by utilizing Agencourt AMPure XP beads (Beckman Coulter, Brea, CA, USA) and then analyzed on a TapeStation 4200 D1000 screentape. Seven hundred fifty nanograms of the gDNA library was prepared for hybridization with the capture baits. The sample was hybridized at 65°C for 24 h, captured with the Dynabeads MyOne Streptavidin T1 (Life Technologies, USA) and purified using Agencourt AMPure XP beads. Use the Agilent protocol to addition of index tags by posthybridization amplification. The sample was sequenced on Illumina NovaSeq with 150PE protocol. The qualified reads data then went through a genomic alignment against Ensembl database using Burrows-Wheeler Aligner (BWA 0.7.15) to get basic sequence information. To further the variants analysis, variants calling and annotations were created by Genome Analysis Toolkit (GATK 3.0) and Variant Effect Predictor (VEP86).

### *In silico* Pathogenicity Assessment of Variants

The potential pathogenicity of the obtained variants was predicted through several online prediction tools such as Sorting Intolerant From Tolerant (SIFT) (http://sift.bii.a-star.edu.sg) (6), Combined Annotation Dependent Depletion (CADD) (https://cadd.gs.washington.edu/) (7), MutationTaster (http://www.mutationtaster.org/) (8), Polymorphism Phenotyping v2 (PolyPhen-2) (http://genetics.bwh.harvard.edu/pph2/) (9), and deleterious annotation of genetic variants using neural networks (DANN) (10). Finally, interpretation of the novel mutation pathogenicity was done using the joint consensus recommendation of the American College of Medical Genetics and Genomics guideline (11).

### Structural Modeling

Homology modeling was performed with SWISS-MODEL (12). The amino acid sequence of basement membrane–specific heparan sulfate proteoglycan core protein was retrieved from UniProt database (http://www.uniprot.org) (accession no. P98160, resides 198-404). The template structure was searched from SWISS-MODEL by using protein sequence BLAST. Top-ranked structure (PDB ID: 1N7D, 48% identity) was selected as the template. Both wild-type and p.Cys375Trp mutant structures were modeled using the same template. Tertiary structures were visualized and compared using the PyMol (http://www.pymol.org) program.

### Primary Cells and Proliferation Assay

The human dermis specimens obtained by skin biopsy were fragmented and laid onto the surface of dishes. The Petri dishes were maintained semiopened in the laminar flow for 40 min in order to adhere the dermis specimens on the dish surface. Then Gibco Dulbecco modified Eagle medium (Thermo Fisher Scientific, Waltham, MA, USA) containing 10% (vol/vol) fetal bovine serum and penicillin/streptomycin was added into the dish. For proliferation assay, cells were grown in a density of 5 × 10^3^/cm^2^. The cell number was counted everyday by using counting chamber.

### Wound Healing Migration Assay

Primary fibroblasts were grown in a 35-mm culture dish with the silicone culture insert until adherence. After washing with phosphate-buffered saline, the insert was removed. Image acquisition was done at 0 and 17 h. The cell-covered area was obtained by the software ImageJ (National Institutes of Health, USA). The change of cell-covered area (%) = (cell-covered area at 17 h – cell-covered area at 0 h)/cell covered area at 0 h.

### Quantitative Dot Blotting and Immunofluorescence

To quantify the levels of secreted perlecan, primary fibroblasts were grown in serum-free medium for 48 h. The conditioned medium was obtained and spotted on the methanol/Tris-buffered saline prewetted nitrocellulose membrane at the center of the assembled well (Hoefer's PR600 Slot Blot Blotting Manifold). After filtering entire samples through the membrane by vacuum, the membrane was washed and blocked by bovine serum albumin/Tris-buffered saline with Tween 20 at room temperature. Afterward, the membrane was incubated with 50 μL primary antibody for 60 min at room temperature, washed three times, and then incubated with 100 μL horseradish peroxidase (HRP)–conjugated secondary antibody (1:3,000, Invitrogen) per well for 60 min. Finally, the HRP signals were visualized by using ECL reagent (Bio-Rad) after washing three times. The primary antibodies used in this study were antiperlecan domain I (1:100, Invitrogen), and antiperlecan domain III (1:500, Invitrogen), and antiperlecan domain V (1:100, Abcam).

For immunofluorescence, primary fibroblasts grown for 5 days were fixed with 3.7% (wt/wt) paraformaldehyde for 30 min. After washing and blocking with serum (Thermo Fisher Scientific, Waltham, MA, USA), cells were labeled with primary antibody overnight at 4°C. Then, cells were stained with fluorescence-conjugated secondary antibodies Alexa Fluor 488–conjugated immunoglobulin G (1:200; Jackson ImmunoResearch Laboratories, West Grove, PA, USA) at room temperature for 1 h after washing. The primary antibodies used in this study were antiperlecan domain I (1:50, Invitrogen), antiperlecan domain III (1:100, Invitrogen), and antiperlecan domain V (1:100, Abcam).

To quantify dot blot and immunofluorescence intensity, all images were obtained at an equal setting of all optical parameters, such as exposure time, gain, and saturation. ImageJ (National Institutes of Health, USA) was used for quantification. Averaged intensity was obtained and corrected with the background of each image. To minimize the confounding effects of proliferation rate between fibroblasts of various individual, intensity of each image was normalized to the final cell counts before obtaining conditioned medium or before fixation.

### Statistical Analysis

Values are presented as mean ± standard deviation. After they passed the Kolmogorov–Smirnov normality test, data were compared by applying Student *t*-tests, one-sample *t*-test, and one- and two-way repeated-measures analysis of variance (ANOVA) using Prism (version 6; GraphPad Software, La Jolla, CA, USA). Differences were considered significant at *P* < 0.05.

## Results

### Genetic Analysis and *in silico* Pathogenicity Assessment

Whole-exome sequencing was performed. After comprehensive annotations of all the variants found, the following genes were particularly analyzed based on the clinical phenotypes: *CBS, CLCN1, COL3A1, COL2A1, COL5A1, COL5A2, COL9A1, COL9A2, COL9A3, COL11A1, COL11A2, EIF4A3, FBN1*, FBN2, *FLNA, HSPG2, LIFR, MED12, MYH11, MYLK, NOTCH1, PHEX, RKG1, POLR1C, POLR1D, SCN4A, SKI, SLC2A10, SMAD3, SMAD4, TCOF1, TGFB2, TGFBR1, TGFBR2*, and *SOX9*. A homozygous NM_005529.6:c.1125C>G; p.Cys375Trp in *HSPG2* gene was identified in both cases. Various *in silico* software applications predicted the mutation to be pathogenic (SIFT, 0; Polyphen-2, 1; CADD, 23.7; MutationTaster, 1; DANN; 0.9). Heterozygous mutation at an identical site was identified in their asymptomatic parents by Sanger sequencing. A diagnosis of Schwartz–Jampel syndrome was made according to the typical clinical features and the autosomal recessive inheritance of the mutant *HSPG2*.

### Structural Modeling

**Figure 3:**
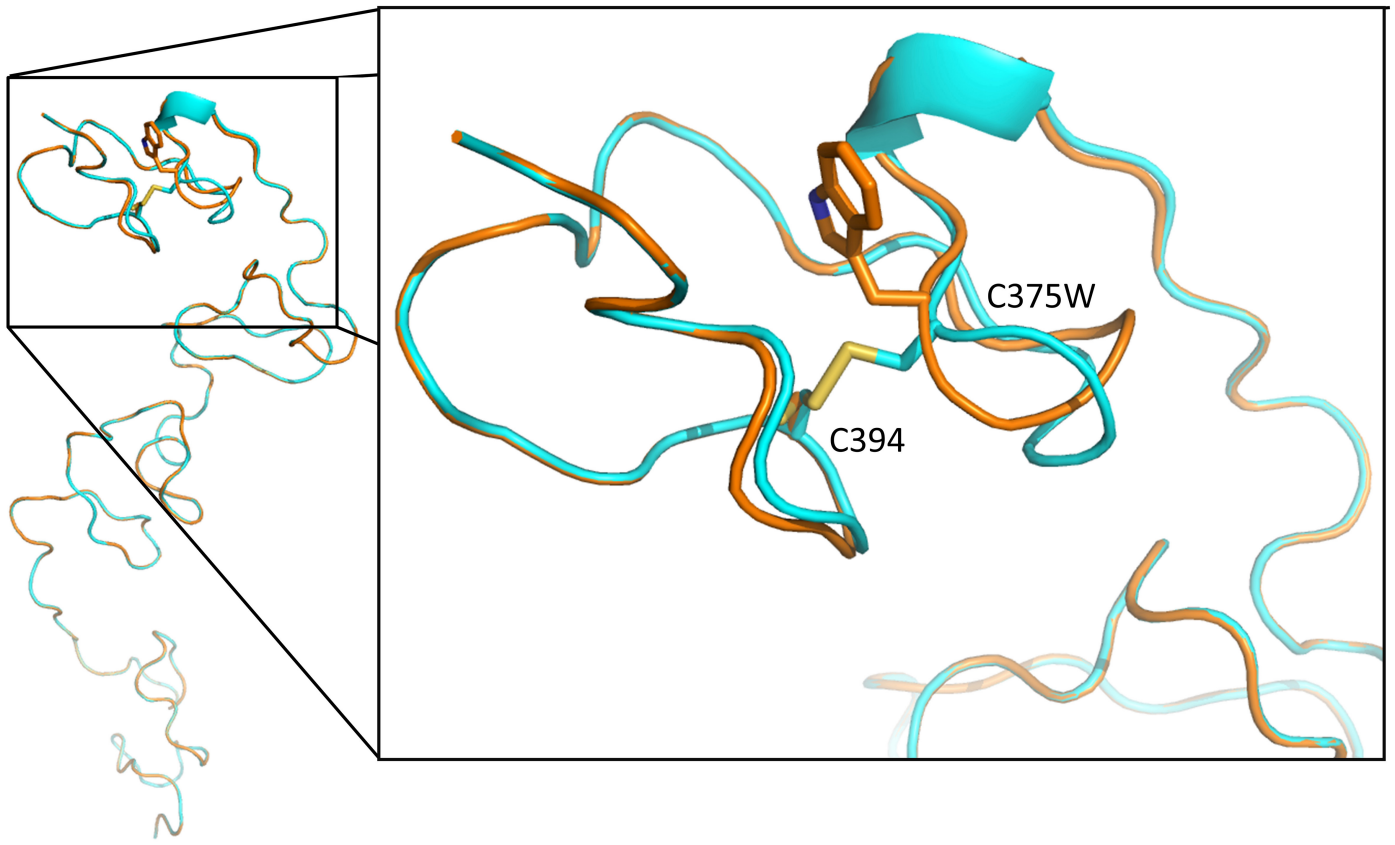
Tertiary structure of LDL-receptor class A 4 domain of perlecan core protein, predicted with SWISS-MODEL. The blue backbone was wild-type tertiary structure, and the orange one is structure with p.Cys375Trp mutation. The mutation leads to loss a disulfide bond between Cys375 and Cys394 (yellow), resulting in loop conformational change and the disruption of this domain structure.

The p.Cys375Trp mutation lies in domain II of perlecan core protein in a low-density lipoprotein (LDL)–receptor class A 4 domain on a loop that is likely stabilized by the disulfide bond between Cys375 and Cys394 (UniProtKB: P98160). The Global Model Quality Estimate score and the *Z* score of qualitative model energy analysis (QMEAN) for SWISS-MODEL were 0.54, −8.4 for wild-type sequence and 0.51, −8.41 for Cys375Trp mutant sequence. The p.Cys375Trp mutation probably leads to the loss of disulfide bond, which may affect the folding of this domain (Figure 3).

### Cellular Function

**Figure 4:**
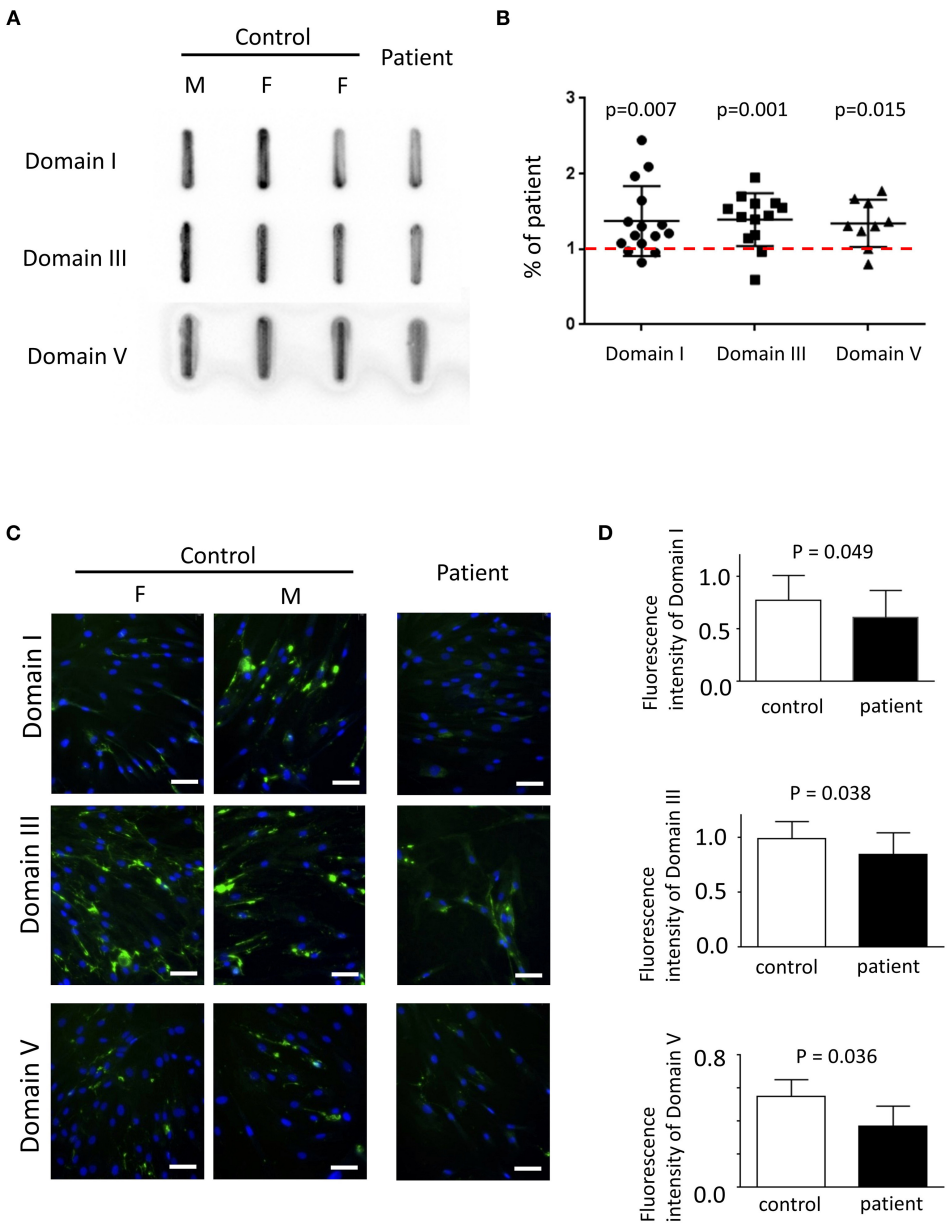
The expression of perlecan in primary fibroblasts. **(A)** Quantitative dot blotting of fibroblast conditioned medium from one patient and three normal subjects (one male and two female subjects). Representative pictures from at least three independent experiments. **(B)** Quantified intensity of each band was normalized to the final cell counts. Dot intensity of all subjects was normalized to the patient. The ratio was compared to one, shown as a dashed red line (one-sample *t*-test). **(C)** Representative pictures of immunofluorescence from one patient and two normal subjects (one male and one female subjects) from six independent experiments (green, perlecan; blue, Hoechst; scale bar, 50 μm; F, female; M, male). **(A,C)** Perlecan was detected by antibody against domain I (top), domain III (middle), or domain V (bottom). **(D)** Comparisons of quantified expression levels of perlecan between control subjects and patients. Data, mean ± SD.

**Figure 5:**
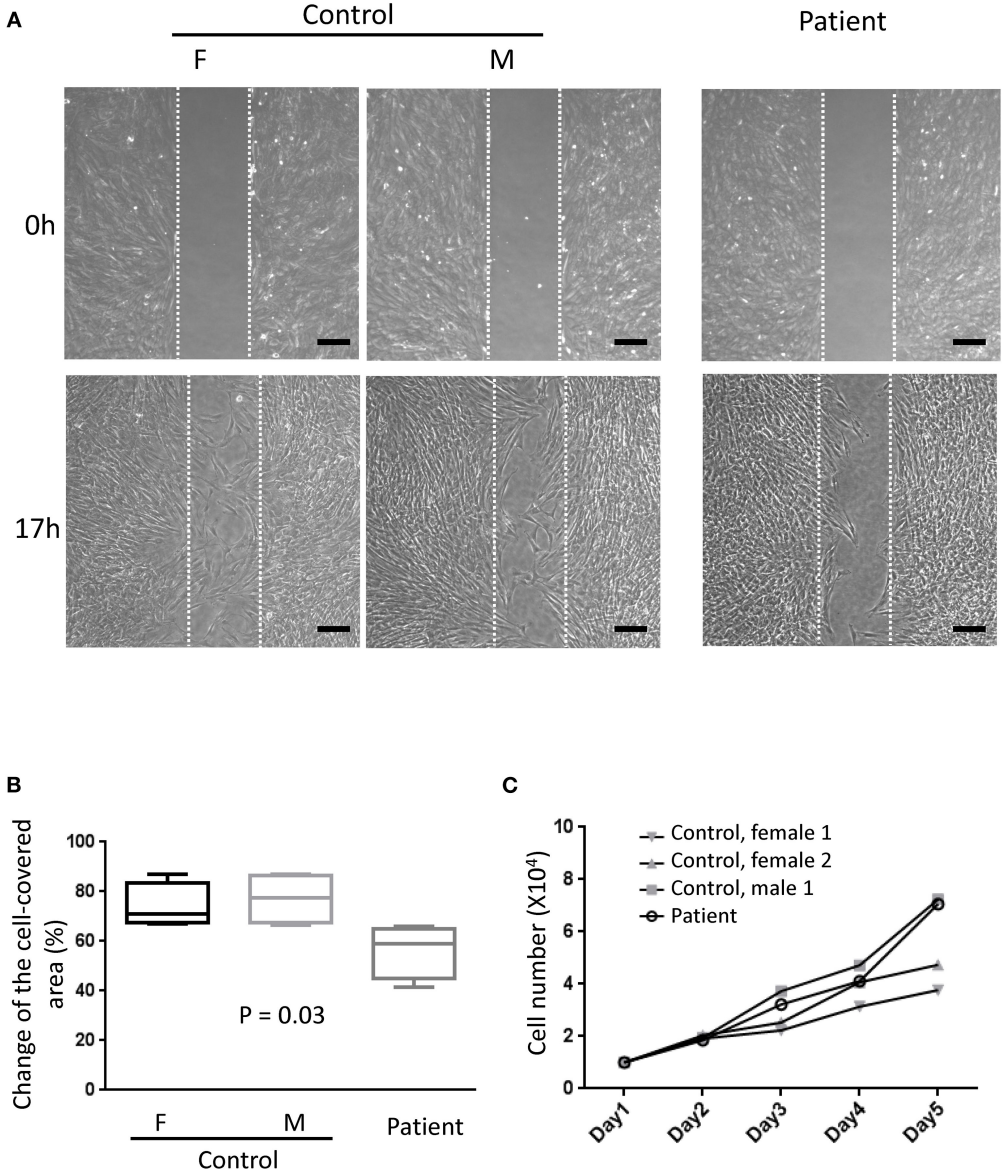
Migration assay. **(A)** Representative photographs from six independent experiments showing cell density at the beginning of removing culture insert and 17 h later. The area between the two parallel white dot lines indicated where the culture insert was (F, female; M, male; scale bar, 200 μm). **(B)** Comparisons of the change of the cell-covered area over time between patients and control subjects. One-way ANOVA, *p* = 0.03. **(C)** The proliferation assays showed no difference between primary fibroblasts from the patient and control subjects. Two-way ANOVA, six independent experiments.

Primary fibroblasts were obtained from three healthy adults (one male subject, aged 27–43 years old) and two patients (one male subject, aged 27–30 years old) via skin biopsy. To quantify the amount of secreted perlecan, quantitative dot blotting for conditioned medium were conducted using antibodies against domains I, III, and V of perlecan (Figure 4A). Fibroblasts obtained from the patient showed a lower level of secreted perlecan (Figure 4B, *P* < 0.05, one-sample *t*-test, all compared with patient sample). To visualize the spatial expression of perlecan, immunofluorescence was conducted after growing fibroblasts for 5 days (Figure 4C). Fibroblasts from the patient expressed less perlecan on and outside cell membrane surface (Figure 4D). The quantified results suggested a significant reduction of expression levels of perlecan in patients (*P* = 0.036–0.049, *t*-test, Figure 4B). Fibroblasts from patients also showed an impaired ability of migration (Figures 5A,B). In a wound healing migration assay, the cell-covered area of patient's fibroblast was lower than that of normal control (*P* = 0.03, one-way ANOVA, Figure 5B). The capability of proliferation was unaffected in patient's fibroblast; Figure 5C).

## Discussion

This study disclosed a novel pathogenic missense mutation of *HSPG2*, which has never been reported before. The mutations of *HSPG2* relevant to Schwartz–Jampel syndrome reported from 1991 to 2020 are summarized in Table 1. This newly identified point mutation was previously registered in a single-nucleotide polymorphism database of the National Institutes of Health as a coding sequence variant as rs543805444. It has never been linked to a clinical pathological condition before. This study demonstrated the pathogenic nature of this mutation site by patient's presentations and functional assay on fibroblast derived from patients.

**Table 1:** Identified sites of *HSPG2* gene relevant to SJS.

**Nucleotide change**	**Peptide change**	**Reference**
c.318+A>G	Not reported	(13)
c.574+481C>T	p.Val192AlafsTer25 (splicing, pseudoexon)	(14)
	Normal product, reduce level	
c.665_675del	p.Arg222GlnfsTer5 (frameshift)	(14)
c.720_1654del	Deletion, undetermined effect	(14)
c.1035G>A	p.Arg3452g (missense)^¶^	(15)
c.1125C>G	p.Cys375Trp (missense)	present report
c.1356-10G>A	Not reported	(15)
c.2746C>T	p.Arg916Ter (nonsense)	(4)
c.2826+15G>A	Splicing, undetermined effect	(4)
c.3056C>T	p.Pro1019Leu (missense)	(14)
c.3263T>C	p.Leu1088Pro (missense)	(16)
c.4432C>T	p.Arg1478Cys (missense)	(14)
c.4473_4475del	p.Leu1491del (deletion)	(14)
c.4595G>A	p.Cys1532Tyr (missense)	(14)
c.4648C>T	p.Arg1550Cys (missense)	(14)
c.4740G>A	Splicing, undetermined effect	(14)
c.4740+5G>A	p.Asp1543_Ser1580del (splicing)	(17)
c.4741-10T>G	Splicing, undetermined effect	(14)
c.5702-5G>A	Splicing, undetermined effect	(18)
c.6179delC	p.Pro2060LeufsTer3 (frameshift)	(14)
c.7006+1G>A	Splicing, undetermined effect	(14)
c.7374+4A>G	Splicing, skip exon 56	(19)
c.7874-2A>G	p.His2624_Val2625ins39 (splicing, insertion)	(14)
	p.Val2625GlufsTer103 (splicing)	
	p.Val2625AlafsTer24 (splicing)	
	Val2625AspfsTer15 (spicing, skip exon 61)	
c.7874 exon fusion 60-61	exon fusion 60-61	(19)
c.8464+4A>G	p.Thr2773ProfsTer25 (splicing)	(14)
c.8544G>A	Splicing, skip exon 64	(19)
c.8680C>T	p.Gln2894Ter (nonsense)	(14)
c.8759-3del9	Splicing, retrain intron 66 or skip exon 67	(19)
c.8788G>A	p.Glu2930Lys (missense)	(20)
c.9181C>T	p.Gln3061Ter (nonsense)	(16)
c.9326delA	p.His3109ProfsTer16 (frameshift)	(14)
	p.Thr3088_His3109del (splicing)	
	p.Asp3065_His3109del (splicing)	
c.9642delC	p.Gln3215LysfsTer7 (frameshift)	(14)
c.10354C>T	p.Arg3452Ter (nonsense)	(14)
c.10355G>A	p.Arg3452Gln (missense)	(14)
c.10776delT	p.Ala3592fsTer6 (frameshift)	(18)
c.10982G>A	p.Arg3661Gln (missense)	(14)
	p.Glu3660GlyfsTer114 (splicing)	
c.11192delG	p.Gly3731GlufsTer30 (frameshift)	(14)
c.11208-7G>A	Splicing, undetermined effect	(14)
c.11671+5G>A	Splicing, undetermined effect	(20)
c.11792_11793insC	p.Leu3932AlafsTer32 (frameshift)	(14)
c.12191delC	p.Pro4065ArgfsTer5 (frameshift)	(14)
c.12899+1G>A	Splicing, undetermined effect	(14)
c.12920del7108	Retain intron 95 or retain intron 94 and 95	(19)

¶ *This was the original description in the reference article, which might be a typing error*.

*HSPG2* is located on chromosome 1p36.1-p35, encoding the core protein of perlecan (21). Perlecan exists in the extracellular matrix of epithelial tissue, endothelial tissue, cartilage, muscle, and bone marrow and is considerably involved in development. Mutations in *HSPG2* are associated with two syndromic disorders. When the mutations are better tolerated, Schwartz–Jampel syndrome occurs. When the mutations are devastating, dyssegmental dysplasia, Silverman–Handmaker type, is developed (22, 23). Until recently, ~150 cases of Schwartz–Jampel syndrome have been reported; however, no clear genotype–phenotype correlation was mentioned (20).

The clinical picture of Schwartz–Jampel syndrome mainly results from neuromuscular hyperactivity and dysplasia in the skeletal system. Neuromuscular hyperactivity leads to muscle cramps, blepharospasm, pursed lip, mask face, and muscle hypertrophy. Developmental skeletal symptoms include short stature, prominent supraorbital ridge, micrognathia, low-set ears, pectus carinatum, kyphoscoliosis, platyspondyly, anisospondyly, coronal cleft and failure of the anterior half of the vertebral body, pes planus, and developmental hip dysplasia (24). Symptoms of other systems included myopia (25), ectopia lentis (26), blepharophimosis, decreased subcutaneous tissue, small testis, malignant hyperpyrexia, intellectual disability, developmental delay, and attention deficit (27).

Perlecan consists of five domains, each carrying out different cellular functions (28). For instance, domain II is involved in calcium signaling, whereas domain V interacts with sodium channels (22). The core protein, with the addition of three to four glycosaminoglycans, forms perlecan, which is one of the largest molecules in the extracellular matrix (29). Because of the very large molecular weight, the knock-in strategy is difficult to apply.

The Cys375 in domain II of perlecan core protein is in an LDL-receptor class A 4 domain, which is predicted to form intramolecular disulfide bond with Cys394. The loss of disulfide bond between Cys375 and Cys394 may lead to the loop conformational change and the disruption of this domain structure (Figure 3). The misfolding may lead to ubiquitination and subsequent degradation of proteins and result in a reduction of secreted perlecan levels as what we found in functional assays of primary fibroblasts (Figure 4).

Perlecan is a ubiquitous component of muscle basement membrane. It is particularly important in neuromuscular junction because of retaining acetylcholine esterase directly, so that insufficient perlecan led to compromised stability of the neuromuscular junction. Perlecan deficiency resulted in the loss of acetylcholinesterase at the motor endplate and cause hyperactivity (3). A chronic deficiency of perlecan led to the remodeling of the neuromuscular junction and presynaptic dysfunction with partial denervation (4). This newly reported pathogenic variant that causes a reduction of perlecan levels (Figure 4) fully supported the basic role of perlecan at neuromuscular junction by demonstrating patient's phenotypes by EMG (Figure 2).

The domain II of perlecan core protein is involved in wingless signaling (22) and calcium signaling (30). The impaired calcium signaling also affects calcium dynamics in endoplasmic reticulum through calcium-ATPase 1 and ryanodine receptor 1 (30). Thus, it is very possible to affect muscle contraction. In addition, perlecan deficiency causes hypertrophy in fast-twitch muscles by reducing myostatin signaling (1) and also can cause atrophy in the slow-twitch muscles, possibly through disinhibition of autophagy related to reduced Akt/mTORC1 signaling (2).

In previous literature, needle EMG often reveals CRDs with higher frequency and lack of typical waxing and waning compared to typical myotonia discharge (24). Mild myopathic MUAPs were also reported (4). Short exercise test and long exercise test were reported to be normal in a previous case report (4), which was different from Fournier pattern I observed in our case. Fournier pattern I was usually related with sodium channel, which interacts with perlecan through domain V (22). However, the pathogenic variant in our case was in domain II and in a previously reported case was in domain III of the perlecan core protein. More studies are needed to address the association between genotypes and phenotypes in short exercise test.

Muscle biopsy revealed cholinesterase deficiency in the neuromuscular junction, denervation of synaptic gutters with reinnervation by terminal sprouting, and long nonmyelinated preterminal nerve segments in a case report (4), which supported our observation and inference of the long-duration MUAPs in our case. Muscular hyperactivity in some patients was reported to be responsive to carbamazepine (31) and procainamide (32).

Taken together, this study demonstrated the pathogenic nature of this newly identified missense mutation by detailed clinical assessments and basic cellular functional assays. Patients with this mutation site showed more neuromuscular instability and relatively mild skeletal abnormality comparing with previously reported cases. Fournier pattern I in short exercise test for myotonia classification was first demonstrated on patients with Schwartz–Jampel syndrome, which may add to the information database of genotype–phenotype correlation for future reference.

## Data Availability Statement

The datasets presented in this study can be found in online repositories. The names of the repository/repositories and accession number(s) can be found here: ClinVar Database (https://www.ncbi.nlm.nih.gov/), NM_005529.7 (HSPG2): c.1125C>G (p.Cys375Trp); ClinVar accession: SCV001399760.1.

## Ethics Statement

The studies involving human participants were reviewed and approved by Institutional Review Board of National Cheng Kung University Hospital. The patients/participants provided their written informed consent to participate in this study. Written informed consent was obtained from the individual(s) for the publication of any potentially identifiable images or data included in this article.

## Author Contributions

P-YL drafted the manuscript. C-KH performed *in silico* pathogenicity assessment and obtained primary skin fibroblast. J-HH collected and analyzed data. Y-TC performed the structural modeling of protein. Y-TS approved the final version of the manuscript on behalf of all authors. All authors contributed to the article and approved the submitted version.

## Conflict of Interest

The authors declare that the research was conducted in the absence of any commercial or financial relationships that could be construed as a potential conflict of interest.

## References

[1] Xu Z Ichikawa N Kosaki K Yamada Y Sasaki T Sakai LY. Perlecan deficiency causes muscle hypertrophy, a decrease in myostatin expression, and changes in muscle fiber composition. Matrix Biol. (2010) 29:461–70. 10.1016/j.matbio.2010.06.001 20541011 PMC2939214

[2] Ning L Xu Z Furuya N Nonaka R Yamada Y Arikawa-Hirasawa E. Perlecan inhibits autophagy to maintain muscle homeostasis in mouse soleus muscle. Matrix Biol. (2015) 48:26–35. 10.1016/j.matbio.2015.08.002 26319110

[3] Arikawa-Hirasawa E Rossi SG Rotundo RL Yamada Y. Absence of acetylcholinesterase at the neuromuscular junctions of perlecan-null mice. Nat Neurosci. (2002) 5:119–23. 10.1038/nn801 11802174

[4] Bauche S Boerio D Davoine CS Bernard V Stum M Bureau C. Peripheral nerve hyperexcitability with preterminal nerve and neuromuscular junction remodeling is a hallmark of Schwartz-Jampel syndrome. Neuromuscul Disord. (2013) 23:998–1009. 10.1016/j.nmd.2013.07.005 24011702

[5] Nicole S Davoine CS Topaloglu H Cattolico L Barral D Beighton P. Perlecan, the major proteoglycan of basement membranes, is altered in patients with Schwartz-Jampel syndrome (chondrodystrophic myotonia). Nat Genet. (2000) 26:480–3. 10.1038/82638 11101850

[6] Ng PC Henikoff S. SIFT: predicting amino acid changes that affect protein function. Nucleic Acids Res. (2003) 31:3812–4. 10.1093/nar/gkg509 12824425 PMC168916

[7] Kircher M Witten DM Jain P O'Roak BJ Cooper GM Shendure J. A general framework for estimating the relative pathogenicity of human genetic variants. Nat Genet. (2014) 46:310–5. 10.1038/ng.2892 24487276 PMC3992975

[8] Schwarz JM Cooper DN Schuelke M Seelow D. MutationTaster2: mutation prediction for the deep-sequencing age. Nat Methods. (2014) 11:361–2. 10.1038/nmeth.2890 24681721

[9] Adzhubei IA Schmidt S Peshkin L Ramensky VE Gerasimova A Bork P. A method and server for predicting damaging missense mutations. Nat Methods. (2010) 7:248–9. 10.1038/nmeth0410-248 20354512 PMC2855889

[10] Quang D Chen Y Xie X. DANN: a deep learning approach for annotating the pathogenicity of genetic variants. Bioinformatics. (2015) 31:761–3. 10.1093/bioinformatics/btu703 25338716 PMC4341060

[11] Richards S Aziz N Bale S Bick D Das S Gastier-Foster J. Standards and guidelines for the interpretation of sequence variants: a joint consensus recommendation of the American College of Medical Genetics and Genomics and the Association for Molecular Pathology. Genet Med. (2015) 17:405–24. 10.1038/gim.2015.30 25741868 PMC4544753

[12] Waterhouse A Bertoni M Bienert S Studer G Tauriello G Gumienny R. SWISS-MODEL: homology modelling of protein structures and complexes. Nucleic Acids Res. (2018) 46:W296–W303. 10.1093/nar/gky427 29788355 PMC6030848

[13] Gurbuz G Albayrak HM. Schwartz Jampel syndrome responding positively to carbamazepine therapy: a case report and a novel mutation. Turk J Pediatr. (2019) 61:967–70. 10.24953/turkjped.2019.06.023 32134596

[14] Stum M Davoine CS Vicart S Guillot-Noel L Topaloglu H Carod-Artal FJ. Spectrum of HSPG2 (Perlecan) mutations in patients with Schwartz-Jampel syndrome. Hum Mutat. (2006) 27:1082–91. 10.1002/humu.20388 16927315

[15] Padmanabha H Suthar R Sankhyan N Singhi P. Stiffness, Facial dysmorphism, and skeletal abnormalities: Schwartz-Jampel syndrome 1A. J Pediatr. (2018) 200:286.e1. 10.1016/j.jpeds.2018.04.077 29866592

[16] Iwata S Ito M Nakata T Noguchi Y Okuno T Ohkawara B. A missense mutation in domain III in HSPG2 in Schwartz-Jampel syndrome compromises secretion of perlecan into the extracellular space. Neuromuscul Disord. (2015) 25:667–71. 10.1016/j.nmd.2015.05.002 26031903

[17] Das Bhowmik A Dalal A Matta D Kandadai RM Kanikannan MA Aggarwal S. Identification of a novel splice site HSPG2 mutation and prenatal diagnosis in Schwartz Jampel Syndrome type 1 using whole exome sequencing. Neuromuscul Disord. (2016) 26:809–14. 10.1016/j.nmd.2016.07.004 27521129

[18] Dai L Fang F Huang Y Cheng H Ren C. Clinical and genetic features of Schwartz-Jampel syndrome in a Chinese child: case report and literature review. Zhonghua Er Ke Za Zhi. (2015) 53:855–9. 10.3760/cma.j.issn.0578-1310.2015.11.012 26758326

[19] Arikawa-Hirasawa E Le AH Nishino I Nonaka I Ho NC Francomano CA. Structural and functional mutations of the perlecan gene cause Schwartz-Jampel syndrome, with myotonic myopathy and chondrodysplasia. Am J Hum Genet. (2002) 70:1368–75. 10.1086/340390 11941538 PMC447613

[20] Yan W Dai J Shi D Xu X Han X Xu Z. Novel HSPG2 mutations causing SchwartzJampel syndrome type 1 in a Chinese family: a case report. Mol Med Rep. (2018) 18:1761–5. 10.3892/mmr.2018.9143 29901129

[21] Kallunki P Eddy RL Byers MG Kestila M Shows TB Tryggvason K. Cloning of human heparan sulfate proteoglycan core protein, assignment of the gene (HSPG2) to 1p36.1–p35 and identification of a BamHI restriction fragment length polymorphism. Genomics. (1991) 11:389–96. 10.1016/0888-7543(91)90147-7 1685141

[22] Martinez JR Dhawan A Farach-Carson MC. Modular Proteoglycan Perlecan/HSPG2: mutations, phenotypes, and functions. Genes (Basel). (2018) 9:556. 10.3390/genes9110556 30453502 PMC6266596

[23] Arikawa-Hirasawa E Wilcox WR Yamada Y. Dyssegmental dysplasia, Silverman-Handmaker type: unexpected role of perlecan in cartilage development. Am J Med Genet. (2001) 106:254–7. 10.1002/ajmg.10229 11891676

[24] Arya R Sharma S Gupta N Kumar S Kabra M Gulati S. Schwartz Jampel syndrome in children. J Clin Neurosci. (2013) 20:313–7. 10.1016/j.jocn.2012.01.057 23219824

[25] Bastola P. Schwartz-Jampel syndrome. Kathmandu Univ Med J. (2010) 8:348–51. 10.3126/kumj.v8i3.6227 22610743

[26] Spranger J Hall BD Hane B Srivastava A Stevenson RE. Spectrum of Schwartz-Jampel syndrome includes micromelic chondrodysplasia, kyphomelic dysplasia, and Burton disease. Am J Med Genet. (2000) 94:287–95. 10.1002/1096-8628(20001002)94:4<287::AID-AJMG5>3.0.CO;2-G 11038441

[27] Viljoen D Beighton P. Schwartz-Jampel syndrome (chondrodystrophic myotonia). J Med Genet. (1992) 29:58–62. 10.1136/jmg.29.1.58 1552548 PMC1015825

[28] Noonan DM Fulle A Valente P Cai S Horigan E Sasaki M. The complete sequence of perlecan, a basement membrane heparan sulfate proteoglycan, reveals extensive similarity with laminin A chain, low density lipoprotein-receptor, and the neural cell adhesion molecule. J Biol Chem. (1991) 266:22939–47. 10.1016/S0021-9258(18)54445-8 1744087

[29] Graham LD Whitelock JM Underwood PA. Expression of human perlecan domain I as a recombinant heparan sulfate proteoglycan with 20-kDa glycosaminoglycan chains. Biochem Biophys Res Commun. (1999) 256:542–8. 10.1006/bbrc.1999.0377 10080934

[30] Pei S Parthasarathy S Parajuli A Martinez J Lv M Jiang S. Perlecan/Hspg2 deficiency impairs bone's calcium signaling and associated transcriptome in response to mechanical loading. Bone. (2020) 131:115078. 10.1016/j.bone.2019.115078 31715337 PMC6945981

[31] Topaloglu H Serdaroglu A Okan M Gucuyener K Topcu M. Improvement of myotonia with carbamazepine in three cases with the Schwartz-Jampel syndrome. Neuropediatrics. (1993) 24:232–4. 10.1055/s-2008-1071547 8232784

[32] Spaans F Wagenmakers A Saris W Reekers A Theunissen P Cremers H. Procainamide therapy, physical performance and energy expenditure in the Schwartz-Jampel syndrome. Neuromuscul Disord. (1991) 1:371–4. 10.1016/0960-8966(91)90124-B 1822347

